# Augmentation of an Engineered Bacterial Strain Potentially Improves the Cleanup of PCB Water Pollution

**DOI:** 10.1128/spectrum.01926-21

**Published:** 2021-12-22

**Authors:** Tomijiro Hara, Yumiko Takatsuka, Eiji Nakata, Takashi Morii

**Affiliations:** a Environmental Microbiology Research Section, Laboratory for Complex Energy Processes, Institute of Advanced Energy, Kyoto University, Uji, Kyoto, Japan; b Institute of Advanced Energy, Kyoto University, Uji, Kyoto, Japan; University of Minnesota

**Keywords:** biphenyl dioxygenase, *bphA*, engineered bacterial strain, *Comamonas testosteroni*, polychlorinated biphenyls, bioaugmentation, oxygen microbubbles, water pollution, composite ratio

## Abstract

Polychlorinated biphenyls (PCBs) are recalcitrant organohalide pollutants, consisting of 209 congeners. PCB cleanup in natural landscapes is expected to be achieved by the metabolic activity of microorganisms, but aerobic PCB-degrading bacteria that inhabit sites polluted by PCBs cannot degrade all PCB congeners due to the specificity of their enzymes. In this study, we investigated the degradability of PCBs when a genetically modified PCB-degrading bacterium was compounded with wild-type PCB-degrading bacteria. We used two bacterial strains, Comamonas testosteroni YAZ2 isolated from a PCB-uncontaminated natural landscape and Escherichia coli BL21(DE3) transformed with a biphenyl dioxygenase (BphA) gene from a well-known PCB degrader, Burkholderia xenovorans LB400. The enzymatic specificities of BphA were 2,3-dioxygenation in the YAZ2 and 2,3- and 3,4-dioxygenations in the recombinant E. coli. For the PCB-degrading experiment, a dedicated bioreactor capable of generating oxygen microbubbles was prototyped and used. The combined cells of the recombinant and the wild-type strains with an appropriate composite ratio degraded 40 mg/L of Kaneclor KC-300 to 0.3 ± 0.1 mg/L within 24 h. All of the health-toxic coplanar PCB congeners in KC-300 were degraded. This study suggested that the augmentation of an engineered bacterial strain could improve the cleanup of PCB water pollution. It also revealed the importance of the ratio of the strains with different PCB-degrading profiles to efficient degradation and that the application of oxygen microbubbles could rapidly accelerate the cleanup.

**IMPORTANCE** PCB cleanup technique in a natural environment relies on the use of enzymes from microorganisms, primarily biphenyl dioxygenase and dehalogenase. Herein, we focused on biphenyl dioxygenase and created a recombinant PCB-degrading E. coli strain. Despite the development of environments for the field use of transgenic microbial strains around the world, verification of the applicability of transgenic microbial strains for PCB cleanup in the field has not yet been reported. We tentatively verified the extent to which degradability could be obtained by an augmentation model of a transgenic strain, the enzyme expression of which is easily regulated in rivers and lakes with PCB pollution. Our experiments used a dedicated bioreactor to model the natural landscape and produced results superior to those of bioremediation or biostimulation methods. The application of micro-nano bubbles, which has recently been discussed, to the cleanup of environmental pollution was also found to be useful in this study.

## INTRODUCTION

Polychlorinated biphenyls (PCBs) are persistent organic pollutants (POPs) (1) and are particularly known for their chemical property of “recalcitrance” (2). PCBs have 10 hydrogen atoms on the biphenyl core replaced by chlorine in various combinations of positions and numbers, producing a maximum of 209 congeners. PCBs tend to become recalcitrant as the number of chlorine substitutions increases.

In 1968, the use of “Kanemi rice oil” that was contaminated with PCBs and used for cooking in Japan caused food poisoning in many people. More than 50 years after this incident, these patients still develop inflammatory diseases such as suppuration (3). Moreover, PCB pollution in natural environments has been confirmed in Japan and worldwide; thus, there are concerns that such pollution could pose a serious threat to human health (4, 5). Consequently, the need to eliminate PCBs from the natural environment is an urgent requirement.

The most recent mechanism for the cleanup of PCBs in natural landscapes is a cooperative reaction between anaerobic reductive dehalogenation and aerobic oxidative degradation (2, 6). The allocation of roles of these cooperative reactions is determined by the depth of the layer of PCB-contaminated soil, sediment, and water (7). Exposure to higher-chlorinated PCB congeners and Aroclor 1242 in aerobic agitated soil slurries led to the selection of the bacterial phyla *Betaproteobacteria* and *Acidobacteria*, which contain most well-known PCB degraders (8). We also reported that aerobic PCB-degrading bacteria are widely distributed from Japan to East Asia, rather than being endemic to a specific region, and their classification ranged from *Betaproteobacteria* and *Gammaproteobacteria* subdivisions to *Actinobacteria* (9). Given this background, we hypothesized that the cleanup mechanisms of PCBs occurring in nature could be artificially recreated.

Prather and Martin (2008) described the development of effective tools for genetic engineering, resulting in the development of microbes that express heterologous biosynthetic pathways and resulting in the production of natural products beyond the genetic confines of the natural host (10). Timmis and Pieper (1999) also described gene technology, combined with a solid knowledge of catabolic pathways and microbial physiology, which enables the experimental evolution of new or improved catabolic activities for such pollutants (11). However, Singh et al. (2015) described many challenges to be addressed concerning the release of genetically engineered bacteria in field conditions. There are significant possible risks linked to the use of genetically engineered bacteria under field conditions, and it is important to address how molecular genetics could contribute to risk mitigation (12).

The degradation mechanisms of aerobic PCB-degrading bacteria have been studied in detail since the discovery of biphenyl-metabolizing bacteria by Lunt and Evans in 1970 (13). Biphenyl dioxygenase, which is involved in the initial reaction of the biphenyl metabolic pathway, plays an important role in the bacterial degradation of PCBs, catalyzing the incorporation of two oxygen atoms into one of the aromatic rings of PCB and inducing phenyl ring cleavage (14). Many studies have used *in vivo* and *in vitro* approaches to the generation of hybrid and mutant dioxygenases (14). These studies have created variants with novel PCB-oxidizing activities, but unfortunately, not all appear to have practical applications.

We produced bacterial cells which overproduced enzymes involved in the degradation of PCB and investigated the specific characteristics of degradation when two or more strains of aerobic bacterial cells of different phyla were mixed. We found that the degradation activity could be relatively easily increased or decreased by the modulation of the conditions of the composite. We also investigated the characteristics of the degradation of PCBs during aerobic cultivation with similar PCB-degrading bacteria, but it was difficult to control the bacterial culture to produce efficient PCB-degrading activities. However, the degradability was dramatically improved by combining a PCB-degrading bacterial strain isolated from the natural environment with a recombinant bacterial strain, which added different enzymatic activity.

In this report, we propose a model of a recombinant PCB-degrading bacterial strain, which has been given different enzymatic activity, augments natural PCB-degrading bacteria widely distributed across the natural environment, and may clean up PCB-contaminated sites with minimal possible risks linked to the use of genetically engineered bacteria in the field. We found that PCBs are rapidly decreased under hyperoxygenated reaction conditions using a microbubble technique.

## RESULTS

### Isolation of aerobic PCB-degrading bacteria and characterization of their biphenyl dioxygenases.

**FIG 1:**
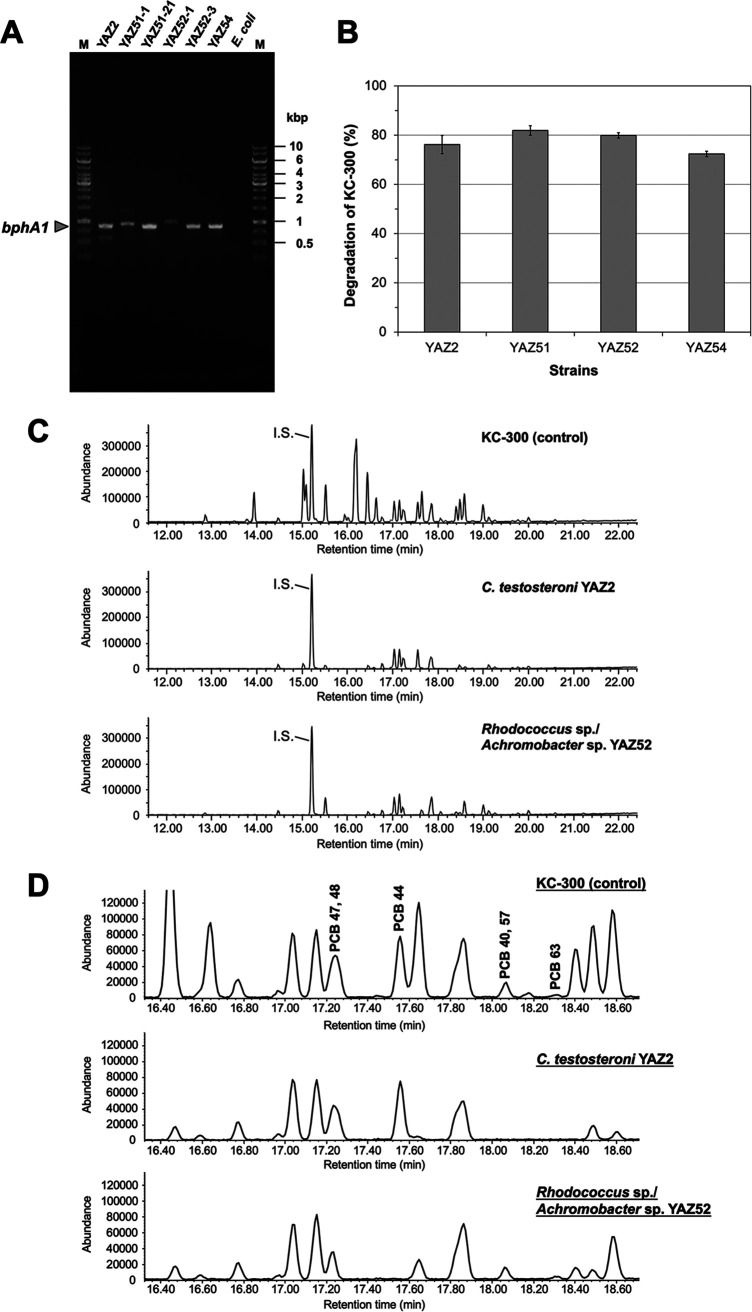
Characteristics of the selected PCB-degrading bacterial isolates. (A) Detection of the gene for the biphenyl dioxygenase large subunit. The strains YAZ2, YAZ51-21, YAZ52-3, and YAZ54 were positively evaluated as containing the *bphA1* gene, which encodes the large subunit of biphenyl dioxygenase, the first and key enzyme of biphenyl and PCB degradation. Sequencing revealed that the PCR product, whose size is close to 900 bp, from nonbiphenyl reactive strain YAZ51-1 encoded not BphA1 but a close homolog of a putative diterpenoid dioxygenase from Pseudomonas pseudoalcaligenes. A faint band approximately 1 kb in size for the nonbiphenyl reactive strain YAZ52-1 was also sequenced and confirmed not to encode BphA1. The E. coli K-12 strain was used as a negative control; M, DNA size marker. (B) Degradation of KC-300 by biphenyl-grown cells of the selected YAZ strains. The reactions were carried out with 5 mg/L KC-300 and the cells of each strain at an OD_660_ of 10 at 30°C for 24 h. The remaining amounts of PCBs were determined using QP-GC/MS, and the percentage degradation of KC-300 was calculated as described in Materials and Methods. The experiment was repeated at least three times, and each reaction was done in triplicate. Error bars show standard deviations; *n* ≥ 3. (C, D) PCB-degrading potency of *C. testosteroni* YAZ2 in comparison with *Rhodococcus* sp./*Achromobacter* sp. YAZ52. The reactions were carried out toward 5 mg/L of KC-300 with the cells of each strain at an OD_660_ of 10 at 30°C for 24 h, and the remaining PCBs were analyzed using QP-GC/MS. (C) Total ion chromatographs (TIC) show the difference in the levels of PCB congener degradation between YAZ2 and YAZ52. (D) TIC profiles show the details of the degradation of the PCB congeners. There were some differences in substrate specificity to PCB congeners between the two strains, even though both strains showed 2,3-dioxygenase activity (see the text for details). YAZ52 data are shown as a representative of the three strains including YAZ51 and YAZ54; 2,2′,4,4′-tetrachrolobiphenyl (PCB 47), which was included in the same peak as PCB 48, was not degraded by any of the four strains when evaluated using a pure reagent (data not shown). I.S., internal standard.

We collected 85 samples from natural landscapes and isolated over 100 aerobic PCB-degrading bacterial strains using enrichment culture with biphenyl as the sole carbon source. We selected four strains—YAZ2, YAZ51, YAZ52, and YAZ54—which had high PCB-degrading abilities, and they were classified as Comamonas testosteroni, *Rhodococcus* sp., Pseudomonas sp., and *Achromobacter* sp. using 16S rRNA gene analysis. The *C. testosteroni* YAZ2 strain showed the same biochemical and PCB-degrading properties as *C. testosteroni* YU14-111, which we previously isolated and characterized (9). It was confirmed that both YAZ51 and YAZ52 comprised two strains, which might have a symbiotic relationship (Table 1). We do not discuss the symbiotic strains in detail here. However, the presence of the biphenyl dioxygenase gene, which plays a crucial role in the first step of PCB degradation, was confirmed in *C. testosteroni* YAZ2 and *Rhodococcus* sp. strains YAZ51-21, YAZ52-3, and YAZ54 by sequencing of the PCR products (Table 1 and Fig. 1A).

**TABLE 1:** Bacterial strains isolated from PCB-uncontaminated environments and used in this study

Strain	Description^*a*^
YAZ2	Comamonas testosteroni, BP^+^
YAZ51	Composed of *Rhodococcus* sp. and Pseudomonas sp., BP^+^
YAZ51-1	Pseudomonas sp., derived from YAZ51, BP^−^
YAZ51-21	*Rhodococcus* sp., derived from YAZ51, BP^+^
YAZ52	Composed of *Rhodococcus* sp. and *Achromobacter* sp., BP^+^
YAZ52-1	*Achromobacter* sp., derived from YAZ52, BP^−^
YAZ52-3	*Rhodococcus* sp., derived from YAZ52, BP^+^
YAZ54	*Rhodococcus* sp., BP^+^

a Isolated strains were identified according to the sequences of the 16S rRNA gene. BP^+^, growth on biphenyl; BP^−^, no growth on biphenyl.

Figure 1B shows the PCB-degrading characteristics of the four strains toward Kanechlor KC-300, in which 50% of congeners contained are 3-chlorine-substituted PCBs (15). In these reactions, bacterial cells grown with biphenyl, and in which the PCB-degrading enzymes were overinduced, were used (see Materials and Methods). The strains YAZ2, YAZ51, YAZ52, and YAZ54 degraded 76.2% ± 3.7%, 81.9% ± 1.9%, 79.9% ± 1.1%, and 72.4% ± 1.1% of PCBs, respectively, toward 5 mg/L of KC-300 in a 24-h reaction.

From the analysis of the degradation profiles of PCB congeners in KC-300, the biphenyl dioxygenases of the four strains were characterized to have 2,3-dioxygenating activity. However, there were some differences between YAZ2 and the other three strains in substrate specificity to PCB congeners. The three strains, except YAZ2, had clearer degrading activities toward 2,2′,4,5-, and 2,2′,3,5′-tetrachrolobiphenyls (PCB 48 and 44) (Fig. 1C and D). YAZ2 clearly degraded 2,2′,3,3′-, 2,3,3′,5-, and 2,3,4′,5-tetrachrolobiphenyls (PCB 40, 57, and 63) (Fig. 1C and D) and also had higher degrading activity toward a pure reagent, 3,3′,4,4′-tetrachrolobiphenyl (PCB 77) (data not shown). These results suggest that the common substrate specificity toward PCB congeners of the three strains, except YAZ2, depends on *Rhodococcus* sp., and also, importantly, that a composite of *C. testosteroni* YAZ2 and any of the other three strains can complement their degradation specificities.

### Optimization of PCB-degrading conditions and regulation by the composite ratio of strains with different substrate specificities.

To evaluate the degradability of PCB, a simple experimental model was constructed assuming water-based bioremediation. We attempted comparative verification between the cases in which microorganisms were introduced with a nutrient supply (the culture model) and those in which bacterial cells were introduced without a nutrient supply (the enzyme model).

**FIG 2:**
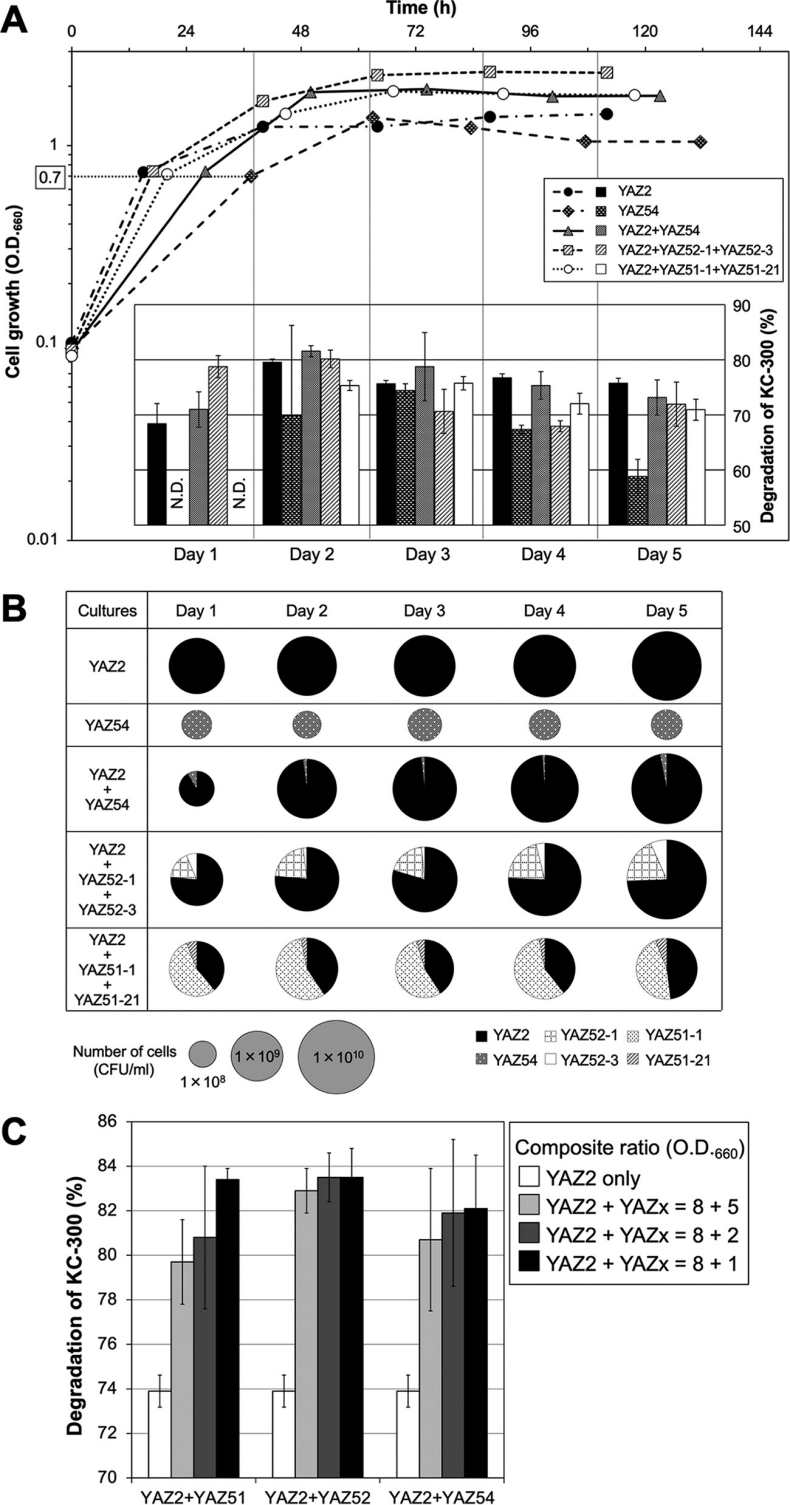
Optimization of the PCB-degrading conditions. (A, B) The culture model. The cells from the mixed cultures could not degrade PCBs efficiently compared with the cells of YAZ2 from the pure culture. (A) The cell growth of the pure cultures (YAZ2 or YAZ54) and the mixed cultures (YAZ2 plus YAZ54, YAZ52-1 and YAZ52-3, or YAZ51-1 and YAZ51-21) in the biphenyl-supplied minimal medium and the percentage of degradation of the 5 mg/L of PCBs for 24-h reactions by the cells collected from the cultures at 1 to 5 days, respectively. The experiment was repeated three times, and PCB-degrading reactions were performed in triplicate. Error bars show standard deviations; *n* = 3. N.D., not detected. (B) The population of each strain in the collected cells from the cultures presented in Fig. 2A; *n* = 3. (C) Complex-response relationship with YAZ2 and other strains for PCB degradation; the enzyme model. Degradation of KC-300 by the complex YAZ2 and YAZ51, YAZ52, or YAZ54 was regulated through modulation of the composite ratios for the strains. The composite ratios at OD_660_ for the strains are indicated in the figure. Error bars show standard deviations; *n* ≥ 3.

Figure 2A shows the cell growth of both pure and mixed cultures of the strains and the PCB degradation by the cells collected from each culture at 1 to 5 days (5-day culture model). The growth of YAZ2 was relatively quick in the biphenyl-supplied minimal medium, and the cells from the pure culture showed relatively constant and high degradation activities in the range of 68.4% ± 3.6% to 79.5% ± 0.6% over the entire culture period. In contrast, the growth rate of the pure culture of YAZ54 was slow, and it took 37.5 h for the optical density at 660 nm (OD_660_) to reach 0.7. The degradation activities of the cells from the YAZ54 pure culture were not stable according to the cell growth. Degradation reached a maximum of 74.4% ± 1.2% at day 3 and then decreased to 58.8% ± 3.1% at day 5. At day 1, no activity was detected. In a mixed culture of YAZ2 and YAZ54, the degradation activities of the cells had a similar tendency to those of a pure culture of YAZ2 with slightly increased activity from day 1 to day 3 (71.0% ± 3.2% to 81.5% ± 1.0%). Although the population of YAZ2 in the collected cells was over 91% through the entire culture (Fig. 2B, YAZ2 + YAZ54), the degradation profiles toward PCB congeners showed that both 2,3-dioxygenases from YAZ2 and YAZ54 were both clearly active at day 2 and day 3 but mostly relied on YAZ54 at day 1 and on YAZ2 at day 4 and day 5, as judged by the degradation of PCB 44, PCB 40, and PCB 57 in KC-300 (data not shown). In a mixed culture of YAZ2 and YAZ52 (*Achromobacter* sp. YAZ52-1 and *Rhodococcus* sp. YAZ52-3 were separately handled for this experiment; see Materials and Methods), the degradation activities were higher than in YAZ2 pure culture at day 1 (78.8% ± 2.0%) and day 2 (80.1% ± 1.6%) when 2,3-dioxygenation from both YAZ2 and YAZ52 was detected and then decreased to lower than that of YAZ2 pure culture from day 3 to day 5, where on the gas chromatography-mass spectrometry (GC-MS) profiles, 2,3-dioxygenase activity only from YAZ2 seemed to be detected (data not shown). The populations of YAZ2, YAZ52-1, and YAZ52-3 in the mixed culture were 75% to 80%, 17% to 20%, and 2% to 7%, respectively, through the entire culture (Fig. 2B, YAZ2 + YAZ52-1 + YAZ52-3). Finally, regarding the mixed culture of three strains, YAZ2, Pseudomonas sp. YAZ51-1, and *Rhodococcus* sp. YAZ51-21, the degradation activity was not detected at day 1, and it was the same or lower than that of the YAZ2 pure culture from day 2 to day 5. The degradation properties were from YAZ2 and YAZ51 at day 2 and after then mainly from YAZ51. The population of the strain YAZ51-1, in which the *bphA1* gene was not detected, was high in this mixed culture from 46% to 58% through 5 days, and those of YAZ2 and YAZ51-21 were 40% to 45% and 3% to 7%, respectively (Fig. 2B). Taken together for the culture model, at some points in some mixed cultures, increased degradation coursed by the complementation of 2,3-dioxygenase specificities was observed; however, it was not stable and it seemed to be difficult to control the degradation efficiently. This culture model did not efficiently degrade PCBs in water-based environments because of the low proliferation of members of the genus *Rhodococcus*.

To examine the enzyme model, bacterial cells of the YAZ strains were collected from the biphenyl-supplied minimal medium, the PCB-degrading enzymes were overinduced in the cells in advance, and the degradation of KC-300 by YAZ strains was evaluated. We initially produced a composite of cells of YAZ2 and the other strains at an OD_660_ ratio of 10 to 10. However, the degradation activity was not significantly greater than that produced by cells of a single strain presented in Fig. 1B, even though the complementation of 2,3-dioxygenase specificities from two mixed strains was observed in some reactions; for example, the degradation of KC-300 by the cells of YAZ2 + YAZ51 at an OD_660_ of 10 to 10 was 76.0% ± 2.4%. It was observed in the culture model that the degradation activities were slightly increased at day 2 of a mixed culture of YAZ2 + YAZ54 in which the population of YAZ2 was much higher than that of YAZ54. Therefore, we decreased the ratio of the three strains including *Rhodococcus* sp. to an OD_660_ of 5, while YAZ2 was kept at OD_660_ of 10, and evaluated the degradation activities toward KC-300. The degradation, compared to the single-strain YAZ2, was increased at an OD_660_ ratio of 10 to 5 by the complex of YAZ2 + YAZ51, YAZ2 + YAZ52, and YAZ2 + YAZ54, to 81.4% ± 0.9%, 83.0% ± 1.3%, and 78.6% ± 3.7%, respectively. The complementation of 2,3-dioxygenase activities from the two mixed strains was clearly observed. The ratio of YAZ51, YAZ52, and YAZ54 was further decreased, and the degradation of KC-300 by the complex with the YAZ2 was evaluated (Fig. 2C). The proportion of YAZ2 was kept at an OD_660_ of 8, and that of the other three strains was at an OD_660_ of 5, 2, or 1. In all of the reactions with the complexed cells, degradation was increased compared with that achieved by single strains of YAZ2, and they increased in accordance with the decrease in the ratio with the other strains (Fig. 2C). The highest degradation was observed at a ratio of YAZ2 to each of the other strains at an OD_660_ of 8 to 1, and the degradation rates were 83.4% ± 0.5%, 83.5% ± 1.3%, and 82.1% ± 2.4%, for YAZ2 + YAZ51, YAZ2 + YAZ52, and YAZ2 + YAZ54, respectively. From the profiles of PCB congeners in the reactions analyzed using GC-MS, the complementation of 2,3-dioxygenase activities from *Comamonas* strain YAZ2 and the other *Rhodococcus* strains was apparent. The degradation could be regulated by modulating the ratios of the strains. This phenomenon was considered to be unrelated to the low proliferative potential of the genus *Rhodococcus*, which was apparent in the culture model, because this result was contrary to the degradation tendencies of the other two models using the genus *Rhodococcus*.

This comparison indicated that the enzyme model was superior to the culture model in terms of degradability, decomposition rate, and manipulability. The use of a composite operation could facilitate the modulation of the degradability.

### Construction of Escherichia coli expressing 3,4-biphenyl dioxygenase and the evaluation of its ability to catalyze PCB congeners.

The biphenyl dioxygenases from all of the isolated PCB-degrading bacteria in this study showed 2,3-dioxygenase activity. To date, it has been clarified that there is 3,4-biphenyl dioxygenase activity for PCB degradation in addition to 2,3-biphenyl dioxygenase activity (2, 16). Therefore, we generated a new engineered E. coli strain expressing 3,4-biphenyl dioxygenase from a Burkholderia xenovorans LB400 strain (17).

**FIG 3:**
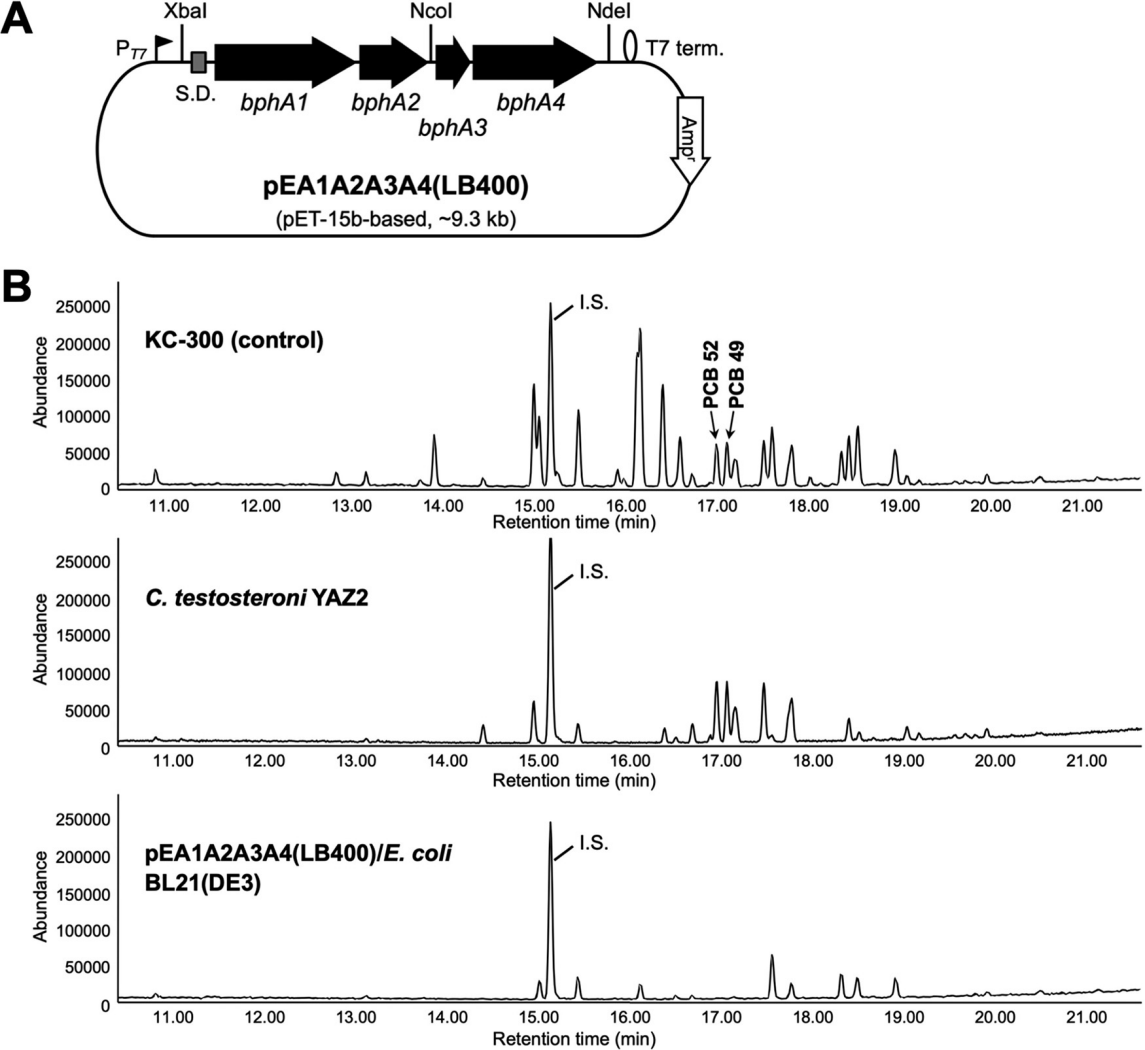
Construction of E. coli expressing 3,4-biphenyl dioxygenase and its ability to degrade PCB congeners. (A) A pET-15b-based 3,4-biphnyl dioxygenase-expressing plasmid was constructed using genome information from *B. xenovorans* LB400. Shine-Dalgarno sequence (S.D.) for *bphA1* used is the original sequence from LB400, not from the pET-15b. T7 term., T7 termination loop. (B) PCB-degrading potency of newly constructed recombinant E. coli expressing 3,4-dioxygenase. Comparison of the TIC from the QP-GC/MS show that E. coli BL21(DE3) harboring pEA1A2A3A4(LB400) efficiently decreased PCB congeners, which were not degraded by the isolated YAZ strains having 2,3-biphenyl dioxygenase.

A plasmid, pEA1A2A3A4(LB400), which expressed 3,4-biphenyl dioxygenase was constructed using a pET-15b plasmid and four genes for the biphenyl dioxygenase subunits, referring to the genome information of B. xenovorans LB400 (Fig. 3A). E. coli BL21(DE3) was transformed with pEA1A2A3A4 (LB400) and evaluated for the degradation of PCB congeners in Kanechlor KC-300. As shown in Fig. 3B, the cells of the recombinant E. coli harboring pEA1A2A3A4(LB400) catabolized 2,2′,5,5′- and 2,2′,4,5′-tetrachlorobiphenyls (PCB 52 and PCB 49), both of which are resistant to the biphenyl dioxygenases from the isolated strains (Fig. 3B and 1C). PCB 52 is a characteristic PCB congener, acting as a substrate for 3,4-biphenyl dioxygenase. The recombinant strain catabolized PCB congeners, such as 2,2′,4,4′-tetrachrolobiphenyl (PCB 47) (Fig. 3B and 1D), showing that it also has 2,3-dioxygenase activity.

### Evaluation of the PCB-degrading ability of a composite microbial model.

In order to examine the best ratio of strains for the effective degradation of PCBs, we first investigated KC-300 degradation by recombinant E. coli and *C. testosteroni* YAZ2 in different ratios with a total OD_660_ of 10. The degradation rate of 5 mg/L KC-300 after 24 h was 97.6% ± 0.1%, 97.0% ± 0.4%, and 94.0% ± 0.6% by the cells of E. coli and YAZ2 in the OD_660_ ratio of 8 to 2, 5 to 5, and 2 to 8, respectively. Since the degradation rate by single recombinant E. coli at an OD_660_ of 10 was 83.1% ± 1.6%, it was clear that the composite microbial catalyst of the generated E. coli and *C. testosteroni* YAZ2 degraded PCBs more strongly than any of the single or combined strains examined in this study (Fig. 1B and Fig. 2C). Given these results, we decided to use E. coli expressing 3,4-biphenyl dioxygenase and *C. testosteroni* having 2,3-dioxygenation activity at the OD_660_ ratio of 8 to 2 (a ratio of 4 to 1) for further experiments.

**FIG 4:**
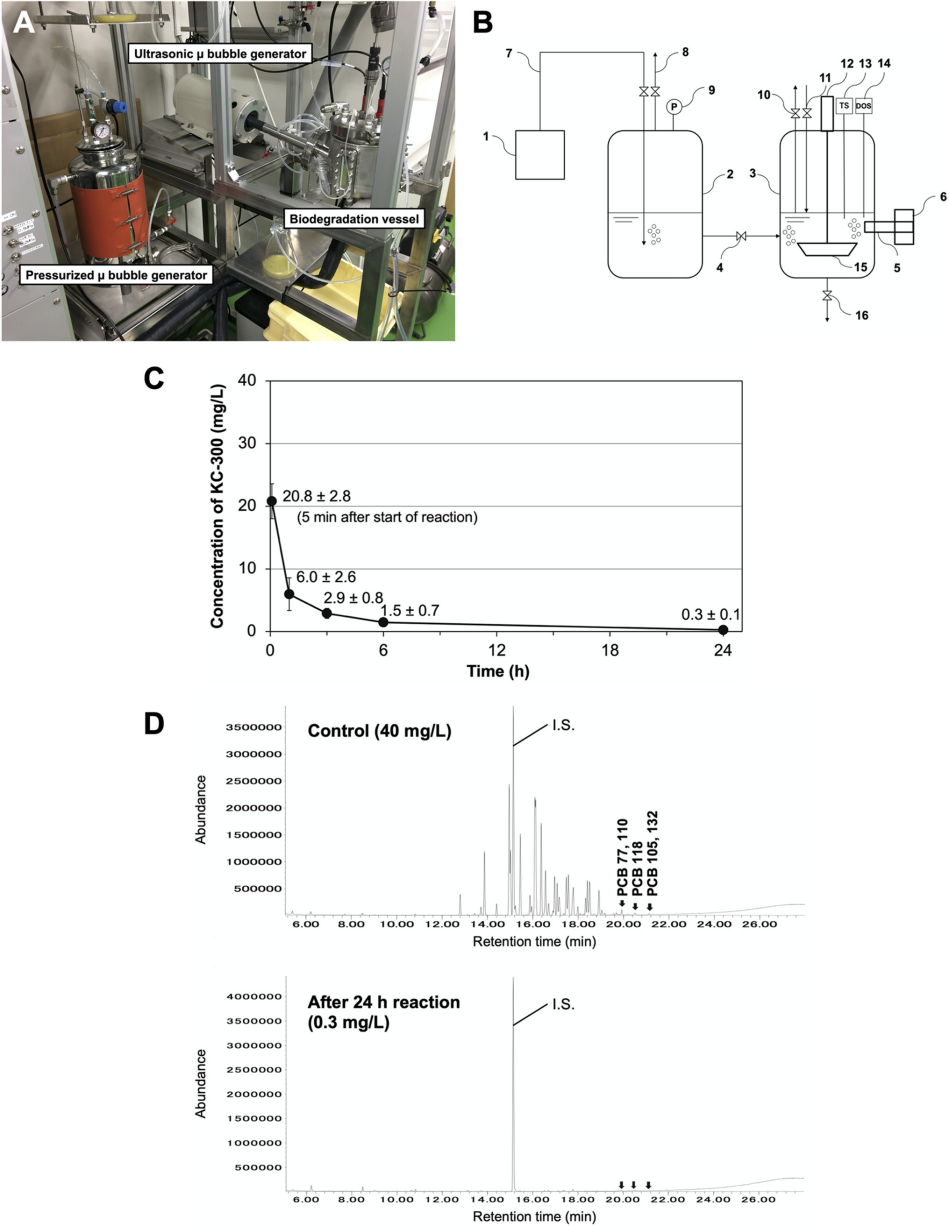
Evaluation of the composite bacterial catalyst using our custom bioreactor with oxygen-supplying microbubbles. (A) The bioreactor used in this study. Oxygen gas required for dioxygenase activity was supplied as bubbles into the buffer in the pressurized-type microbubble generation tank in advance. Then, the buffer with generated oxygen microbubbles was transferred to the 2-L scale of the biodegradation vessel. PCBs and bacterial catalysts were added directly into the vessel through the sample injection valve system. (B) Schematic drawing of the bioreactor. The features of the bioreactor are as follows: 1, oxygen-gas cylinder unit; 2, pressurized-type microbubble generation tank; 3, biodegradation vessel; 4, pressurized-water (solvent) release valve; 5, ultrasonicated-type microbubble generator nozzle; 6, ultrasonic oscillator; 7, oxygen-gas pipeline; 8, oxygen-gas release valve system; 9, pressure gauge (MPa); 10, sample collection valve system; 11, sample injection valve system; 12, stir bar rotary power motor; 13, temperature and pH sensor; 14, dissolved oxygen meter; 15, paddle; 16, drain valve. (C) Time-dependent PCB degradation by the composite bacterial catalyst in the bioreactor. Kanechlor KC-300 (40 mg/L) was degraded to 0.3 ± 0.1 mg/L after 24 h of reaction. The reaction conditions were as follows: reaction volume of 1 L, temperature of 30°C, and the bacterial strains used as catalyst at a total OD_660_ of 20 with the ratio of the recombinant E. coli and *C. testosteroni* YU14-111 at 4:1. Error bars denote standard deviations; *n* = 3. (D) TIC profiles from GC-MS analysis of the reaction mixture after 24 h. Kanechlor KC-300 is composed of about 70 PCB congeners. Almost all congeners, including highly toxic coplanar PCBs, were efficiently degraded by the composite bacterial catalyst with 2,3- and 3,4-biphenyl dioxygenases after 24 h of reaction. Panel D is modified from Fig. 5 in reference 42.

We produced a biological reactor dedicated to a model experiment to clean up PCB water pollution by augmentation of the engineered bacterial strain (Fig. 4A). Two stainless steel water tanks were mounted on a biological reactor, and both tanks were connected by piping. One was a 2-L biochemical reaction tank that could be adjusted to an appropriate temperature, and the other was a pressurized microbubble generation tank. The biological chemical reaction tanks were equipped with several sensors to monitor dissolved oxygen, pH, and temperature (Fig. 4B).

In this experiment, two biphenyl dioxygenase-overexpressing bacterial strains were prepared in 1 L of the reaction solution at a total OD_660_ of 20, and the strain having 2,3-dioxygenation activity *C. testosteroni* YU14-111 was used. *C. testosteroni* YU14-111 displayed the same biochemical and PCB-degrading properties as *C. testosteroni* YAZ2 (9). The purpose of this strain alternation was to determine if different strains with the same biochemical and PCB-degrading properties would exhibit similar PCB biodegradability. The composite ratio of the E. coli BL21(DE3) harboring pEA1A2A3A4(LB400) to *C. testosteroni* YU14-111 was adjusted to 4 to 1, producing an OD_660_ of 16 to 4. This experiment showed that the composite microbial model rapidly catabolized 40 mg/L of Kanechlor KC-300 to 0.3 mg/L within 24 h (Fig. 4C). The composite microbial strains also eliminated all coplanar PCB congeners in Kanechlor KC-300 (PCB 77, 118, and 105) (Fig. 4D).

The degradation potencies of the composed bacterial catalyst toward the PCB Kanechlor series were also examined using 5 mg/L for each of Kanechlor KC-300, KC-400, KC-500, and KC-600 in a small-scale reaction in glass vials for 24 h. As the bacterial catalyst, the cells of E. coli BL21(DE3) harboring pEA1A2A3A4(LB400) and *C. testosteroni* YAZ2 were used at an OD_660_ of 8 + 2. Low-chlorinated congener-dominant PCBs, KC-300 and KC-400, were almost completely degraded, and the degradation rates were 97.6% and 93.4%, respectively. The degradation rates of KC-500 and KC-600, in which those high-chlorinated PCBs were dominant, were low at 67.4% and 46.1%, respectively.

## DISCUSSION

In this study, we tried to engineer one aerobic bacterial strain to supplement wild-type aerobic PCB-degrading bacteria occurring in natural landscapes in Japan, to supplement their insufficient cleanup ability. The results of all experiments were judged according to whether the cleanup ability improved.

The wild-type aerobic PCB-degrading bacterial strains were isolated from landscapes uncontaminated by PCBs. When PCB-degrading bacteria are placed in a specific ecological niche, they acquire genes necessary for metabolism by horizontal gene transfer from other, similar, bacteria (18, 19). Therefore, we had to obtain unspecified and unmutated PCB-degrading bacteria from environments uncontaminated with PCBs. Candidate strains of aerobic PCB-degrading bacteria isolated from environments uncontaminated with PCBs were identified as *C. testosteroni*, *Rhodococcus* sp., *Achromobacter* sp., and Pseudomonas sp. using 16S rRNA gene analysis (Table 1 and Fig. 1A). We found a strain in a genus similar to the aerobic PCB-degrading bacteria reported so far (16, 20–23).

We did not focus only on the ecology of aerobic PCB-degrading bacterial strains but also considered the substrate specificity of biphenyl dioxygenases (BphA), which catabolize PCB congeners and are important factors in the trial of this bioaugmentation model. There have been many reports on the substrate specificity of BphA for PCBs, and there have been multiple attempts to genetically modify the enzyme (24–28). The substrate specificity of BphA for PCB congeners can be roughly divided into two types of biphenyl dioxygenase activities; one is a biphenyl-3,4-dioxygenase activity from a B. xenovorans LB400 strain, and the other is a biphenyl-2,3-dioxygenase activity from another aerobic PCB-degrading bacterial strain, including the LB400 strain. We confirmed, using PCR, that aerobic PCB-degrading bacterial strains obtained from the natural landscape around Yonezawa city conserve the *bphA* gene (Fig. 1A). The substrate specificity of BphA for PCBs was also investigated using a microbial catalyst, which overexpressed *bph* genes, resulting in biphenyl-2,3-dioxygenase activity (Fig. 1C). Isolated PCB-degrading bacteria from the natural landscape, which were used as controls in this study, did not have to be unique and had to be from a genus that could be easily found.

We constructed experimental models to investigate the effective use of wild-type PCB-degrading bacterial strains in the cleanup of PCB. These models were assumed to be comparable to the bioaugmentation method (enzyme model) (Fig. 2C) toward the biostimulation method (culture model) (Fig. 2A and B). The results suggested that the bioaugmentation method was superior in terms of degradability and regulatability. Strains in which the PCB-degrading enzyme was overinduced in advance were used as the wild-type PCB-degrading bacteria in the bioaugmentation method. Consequently, we assumed that the “amount of PCB-degrading enzyme” was important. Furthermore, when we attempted to degrade PCBs using a composite of two types of enzyme-overinduced strains with different PCB substrate specificities, we found that PCB degradability differed depending on the ratio (amount) of the composited enzymes (Fig. 2C). This result suggests that the following two factors are important in addition to the amount of PCB-degrading enzyme: “oxygen consumption” and a “combination of substrate specificity.”

In the case of Kanechlor KC-300 as PCB, the degradation rates produced by PCB-degrading bacterial strains isolated from the natural landscapes ranged from 72.4% ± 1.1% to 81.9% ± 1.9% (Fig. 1B). These degradation rates were not sufficient to clean up PCB pollution. Therefore, we attempted to improve the degradation rate by forcing not only the biphenyl-2,3-dioxygenase activity shown by the isolated strains but also the biphenyl-3,4-dioxygenase activity shown by the LB400 strain (16). BphA determines the range of PCB congeners oxidized and plays an essential role in substrate recognition and binding (29). Genetically modified BphA containing a motif of the BphA gene of the LB400 strain has been investigated in detail (26–28). We, therefore, constructed a recombinant PCB-degrading bacterium that produced the BphA of the LB400 strain using E. coli as a host strain and confirmed that its substrate specificity was biphenyl-3,4-dioxygenase (Fig. 3A and B). By implementing mutant BphA showing various substrate specificities in transgenic PCB-degrading bacterial strains, it will be possible to evaluate the cleanup ability of PCB pollution compared to wild-type PCB-degrading bacteria as a natural landscape model. It is possible that the enzymatic activity significantly differed between cells in the frozen stock and those in the fresh culture. A significant difference was observed for the recombinant strain. However, no significant difference was observed with respect to degradability among the wild-type strains. Therefore, in this study, wild-type strains were used as frozen stocked cells, and the recombinant strain was used as fresh cultured cells (all data not shown).

We produced a prototype bioreactor to embody rivers, lakes, and marshes in a natural landscape model (Fig. 4A and B). The reaction vessel of this device is equipped with a paddle so that a natural flow velocity can be applied, and the water temperature can be adjusted. In addition, one unique function was provided: it was possible to create hyperoxygenated reaction conditions by generating microbubbles in the solvent. In recent bioremediation technologies, the application of micro-nano bubble technology has begun to be discussed (30–32), and we argue in this study that this technology is also useful for the cleanup of PCBs. Biphenyl dioxygenase as BphA introduces two oxygen atoms into PCB, requiring a sufficient amount of oxygen molecules. The wild-type aerobic PCB-degrading bacterial strain *C. testosteroni* YU14-111, which we isolated from the natural landscape, reduced 10 mg/L and 100 mg/L PCB waste oil by 65.8% ± 4.7% and 62.6% ± 9.9%, respectively. However, when oxygen microbubbles were introduced, the degradability increased to 71.6% ± 3.7% and 70.7% ± 10.1%, respectively (data not shown).

Using the bioreactor, we attempted a degradation experiment in which a genetically modified PCB-degrading E. coli strain was compounded with a wild-type aerobic PCB-degrading bacterial strain. In the OD_660_ value, the ideal composite ratio of the recombinant type was 4, and that of the wild-type was 1. Originally, enzyme activity must be expressed as a specific activity value and not an OD value. However, as a result of examining the specific activity value using nonchlorinated biphenyl as a standard substrate, the value could not be obtained because the catabolism reaction was too fast. As a result of this experiment, 40 mg/L Kanechlor KC-300 was degraded to 0.3 ± 0.1 mg/L within 24 h. All of the health-toxic coplanar PCBs (PCB 77, 118, and 105) in the KC-300 were completely degraded (Fig. 4C and D).

Overall, this study suggested that by adding a strain of E. coli in which PCB degradability was strengthened by gene recombination technology to aerobic PCB-degrading bacteria those are abundant in the natural environment, Kanechlor KC-300 and KC-400 included PCBs can be cleaned up extremely efficiently compared to commonly adopted bioremediation methods. The associated result can be found in the [Results] section. The application of oxygen microbubbles assisted in rapid cleanup.

There are several advantages of our study over previous studies. Previous research has been restricted to characterizing PCB-degrading bacterial strains (13, 23, 33, 34), the substrate specificity (2, 14, 16, 35) or genetic modifications (27, 36, 37) of biphenyl dioxygenase, and investigating PCB-degrading bacterial flora in PCB-polluted natural landscapes (8, 38–40). Herein, we were able to identify the application model in which the newly constructed recombinant E. coli expressing biphenyl dioxygenase of the *B. xenovorans* LB400 strain was augmented to the isolated wild-type PCB-degrading bacterial strain. In addition, we developed the bioreactor dedicated to this application model that reproduces a polluted water environment model. This provided us with the advantage of conducting a more specific assessment of environmental water remediation. Hence, we have shown the potential for the efficient cleanup of PCBs from water environments by augmenting recombinant strains to wild-type PCB-degrading bacteria, which were already habituated to natural landscapes. Furthermore, the hyperoxygen supplying method involving the microbubble technique may be a promising method for activating many types of dioxygenases involved in pollutant degradation. It is possible that environmental bioremediation research involving recombinant strains based on synthetic biology will soon become more prevalent. Therefore, the biological reactor system could potentially be used as a preliminary evaluation method in such research. Finally, we confirmed that the efficiency of PCB degradation under aerobic conditions is affected by both the ratio of strains used and the use of oxygen microbubbles. Thus, we argue that this finding validates the effectiveness of the developed method for efficient remediation of pollution containing PCBs equivalent to Kanechlor KC-400 (products containing 50% 4-chlorinated PCBs) from the water environment.

In brief, we plan to report new or improved catabolic activity toward PCB pollutants based on genetic technology combined with a solid knowledge of catabolic pathways and microbial physiology, as described by Timmis and Pieper (11). We will continue to study natural landscape models that efficiently clean highly chlorine-substituted PCBs by creating artificially enhanced PCB-degrading microbial strains aimed at experimental evolution.

## MATERIALS AND METHODS

### Chemicals.

The PCBs Kanechlor KC-300, KC-400, KC-500, and KC-600 were purchased from GL Sciences Inc. (Tokyo, Japan), and PCB congeners were purchased from AccuStandard Inc. (New Haven, CT, USA). PCBs were dissolved in dimethyl sulfoxide (DMSO) or ethyl acetate at concentrations of 1 to 10 mg/mL to prepare stock solutions and further diluted with DMSO if necessary. For Kanechlor, the digit in “100” beginning behind “KC-” shows the substituted chlorines number of the primary ingredient contained in 50% or more of the whole PCBs; in brief, the case of the “KC-300” shows that it contains 50% or more of the PCBs of the 3-chlorine substitutions. For example, the case of the KC-300 explains that it contains 50% of the PCB congeners of the 3-chlorine substitutions. The higher that number, the higher the content of PCB congeners with a large number of chlorine substitutions (15). Other general reagents were obtained from Fujifilm-Wako Pure Chemical Corp. (Osaka, Japan). Restriction enzymes for DNA manipulations were purchased from TaKaRa Bio Inc. (Shiga, Japan).

### Isolation of aerobic PCB-degrading bacteria.

Environmental samples were collected from various PCB-uncontaminated sites in and around Yonezawa city. Biphenyl-utilizing bacterial strains, including YAZ2, YAZ51, YAZ52, and YAZ54, were isolated from the environmental samples by repeated enrichment cultures using W-minimal medium, which had the same composition as the mineral salts medium (41), supplemented with biphenyl as a sole carbon source. Biphenyl was added at a concentration of 0.1% for liquid medium and for agar plates given as vapor on the lid of petri dishes subsequently sealed with tape. Enrichment culture was performed at 30°C under three different conditions, in which the pH of the W-medium was adjusted to 4.5, 7.5, or 8.5, respectively. Single colonies grown on W-medium plates were selected, and cell cultures cultivated in W-medium (such as strains YAZ2, YAZ51, and YAZ52) or in Luria-Bertani (LB) medium (1.0% Bacto tryptone, 0.5% Bacto yeast extract, 0.5% NaCl) (such as strain YAZ54) with biphenyl were stored at −80°C in 15% to 18% glycerol.

The strains YAZ51 and YAZ52 were composed of two strains, and further isolation was carried out using a 1/5 LB medium (0.2% Bacto tryptone, 0.1% Bacto yeast extract, 0.5% NaCl) plate with biphenyl vapor. The strains YAZ51-1 and YAZ51-21 from YAZ51 as well as YAZ52-1 and YAZ52-3 from YAZ52 were isolated as single colonies, and each cell culture was stored at −80°C in glycerol.

A heat extract containing genomic DNA from each isolated strain was used as a PCR template. Briefly, bacterial cells were suspended in Tris-EDTA (TE) buffer (10 mM Tris-HCl, 1 mM EDTA, pH 8.0), heated at 98°C for 20 min, and then centrifuged. The supernatant was then collected and used for PCR. Approximately 1,500 bp of the partial 16S rRNA gene was amplified using the primers Bact-8F (5′-AGAGTTTGATCCTGGCTCAG-3′) and Bact-1492R (5′-GGCTACCTTGTTACGACTT-3′). The resulting fragment was purified and directly sequenced using the primers 8F, 1492R, and/or 907R (5′-CCCCGTCAATTCCTTTGAGTTT-3′) via the sequencing service of Fasmac Co., Ltd. (Kanagawa, Japan). DNA sequences were analyzed using the BLAST tool of the National Center for Biotechnology Information to determine their phylogenetic affiliations.

The biochemical characteristics of the isolated strains were examined, if necessary, using an API kit for bacterial identification from bioMérieux (Lyon, France) according to the instructions provided by the company.

### Detection of the gene for the biphenyl dioxygenase large subunit.

The isolated strains were evaluated for whether they contained the *bphA1* gene encoding the biphenyl dioxygenase large subunit. Cells of each strain were suspended in TE buffer, and after heating at 98°C for 20 min, the cellular debris was removed by centrifugation. The supernatant containing DNA from the cells was used for the amplification of *bphA1* by PCR, using the degenerate primers BphA(uni)-99F (5′-AAC(C/T)(A/C)(C/G)TG(C/T)(A/C)GICA(C/T)CG(A/T/G/C)GG(A/T/G/C)ATG-3′, in which I represent inosine) and BphA-390R (5′-TT(C/T)TC(A/G/T)CC(A/G)TCGTCCTGCTC-3′) at an annealing temperature of 60°C. These primers were designed at two highly conserved positions in the amino acid sequences of BphA1 subunits from Comamonas testosteroni YU14-111 (GenBank accession no. BAM05536), *Acidovorax* sp. KKS102 (GenBank accession no. BAJ72245), Pseudomonas pseudoalcaligenes KF707 (GenBank accession no. AAA25743), Burkholderia xenovorans LB400 (GenBank accession no. AAB63425), Rhodococcus jostii RHA1 (GenBank accession no. BAA06868), Rhodococcus erythropolis TA431 (GenBank accession no. BAF48503), and *Bacillus* sp. JF8 (GenBank accession no. BAC79226). The resultant PCR product of around 900 bp, which covers about 65% of the entire *bphA1* gene, was sequenced to confirm that it encodes BphA1.

### PCB degradation assay employing bacterial cells and GC-MS analysis.

Each strain was precultured in 2 mL of W-medium containing 0.1% biphenyl and then transferred to 10 mL of W-medium with biphenyl and grown to an OD_660_ of >0.5 at 30°C. The cells were then harvested at 15°C and used immediately or stored at −80°C until use. For the selected strains, YAZ2, YAZ51, YAZ52, and YAZ54, the cells were further grown in 3 L of W-medium using a jar fermentor BMS-03NC (ABLE corp., Tokyo, Japan) under the following conditions. Biphenyl was added five times every 2 h at a final concentration of 0.02% each time, and for the strains YAZ51, YAZ52, and YAZ54, 0.01% yeast extract was also added at the beginning of the culture. After the culture reached an OD_660_ of 1.0, cells were collected by centrifugation, washed with 20 mM sodium phosphate buffer at pH 7.5 (buffer A), and stored at −80°C.

For the PCB degradation assay, the frozen cells were resuspended and washed twice with buffer A. The reaction was completed in a 4-mL borosilicate glass vial sealed with Teflon-covered packing (As One Corp., Osaka, Japan). The final concentration of cells was adjusted to an OD_660_ of 5 to 60 in a reaction mixture with a total volume of 0.5 mL. Kanechlor KC-300 or a mixture of KC-300 and KC-500 (1:1) was added at final concentrations of 5 to 100 mg/L. If necessary, Triton X-100 was added to a final concentration of 0.005%, especially when the reaction was performed at a high PCB concentration (e.g., at 100 mg/L). A control reaction mixture, in which the buffer was added instead of the cell suspension, was prepared for each reaction. Reaction mixtures were incubated in glass vials for 24 h at 30°C with rotation at 50 rpm produced by a HulaMixer (Thermo Fisher Scientific KK, Tokyo, Japan).

After the reaction was stopped by adding 20 μL of 1 or 5 N HCl, anthracene dissolved in toluene was added as an internal standard at a concentration of 1.6 to 32 mg/L depending on the initial PCB concentration. The remaining PCBs were then extracted twice with 1 mL of ethyl acetate for each extraction. Approximately 2 mL of ethyl acetate extract was dehydrated with sodium sulfate, and the PCBs were analyzed using gas chromatography-mass spectrometry (GC-MS) (model 7890A/5975C; Agilent Technologies, Santa Clara, CA, USA) with an HP-5ms column (30 m by 0.25 mm by 0.25 μm). Helium was used as the carrier gas at a flow rate of 1.5 cm/s. The column temperature for GC was as follows: 80°C for 2 min, which was increased to 130°C at the rate of 20°C/min, followed by heating to 300°C at 8°C/min. A quadrupole detector was used with the following temperatures for the ion source, quadrupole, and transfer line: 230°C, 150°C, and 300°C, respectively. Data acquisition was performed using MSD ChemStation software (Agilent Technologies). Compounds were identified using the NIST 08 Mass Spectral Library for confirmation. The percentage degradation of each reaction mixture was calculated by dividing the concentration of PCBs from the reaction mixture by the concentration of PCBs in the control mixture (without bacterial cells), multiplying the result by 100, and subtracting the resulting value from 100.

### PCBs degradation assay using cells from multiple-strain cultures.

The single-strain and multiple-strain cultures were carried out as follows. The cells were precultured at 30°C in the W-medium with 0.1% biphenyl and 0.01% yeast extract for 16 to 48 h for the strains YAZ2, YAZ54, YAZ52-3, and YAZ51-21, or in 1/5 LB medium with 0.05% biphenyl for 24 h for the strains YAZ52-1 and YAZ51-1. For the single-strain cultures, the precultured cells of YAZ2 or YAZ54 were inoculated into 150 mL of W-medium with 0.1% biphenyl in an Erlenmeyer flask of 1-L volume size with sidearms at an OD_660_ of around 0.1. The inoculated cell numbers were 5 × 10^9^ for YAZ2 and 4 × 10^7^ for YAZ54. For the multiple-strain cultures, the same numbers of the cells of two or three individual strains were inoculated into 150 mL of W medium with 0.1% biphenyl at a final OD_660_ of about 0.1. The inoculated cell numbers were 4 × 10^7^ each for the multiple-strain cultures of YAZ2 + YAZ54, 1 × 10^9^ each for the culture of YAZ2 + YAZ52-1 + YAZ52-3, and 4 × 10^8^ each for the culture of YAZ2 + YAZ51-1 + YAZ51-21. All single- and multiple-strain cultures were carried out at 30°C with shaking at 110 rpm, and once the OD_660_ reached 0.7, the cells from 30 mL of each culture were harvested and stored at −80°C (the cells of day 1). Every 24 ± 2 h after the first sampling, the cell harvest was repeated four times, and the cell samples were stored at −80°C (the cells of day 2 to day 5). All frozen-cell samples were analyzed for PCB-transforming activity as described above. When the cells were harvested, the culture suspensions were diluted and plated onto 1/5 LB plates to analyze the CFU and population of each strain. Experiments were repeated at least three times to confirm the reproducibility of the data.

### Construction of the plasmid pEA1A2A3A4(LB400) for expression of 3,4-biphenyl dioxygenase from *B. xenovorans* LB400.

In order to obtain 3,4-biphenyl dioxygenase, the plasmid to express the biphenyl dioxygenase from *B. xenovorans* LB400 that was reported to have both 2,3- and 3,4-dioxygenase activities was constructed. Two DNA fragments, one of 2,120 bp containing *bphA1A2* and its flanking region and one of 1,600 bp containing *bphA3A4* and its flanking region, were chemically synthesized using the genome sequence information of *B. xenovorans* LB400 (GenBank accession no. NC_007953.1) via the DNA synthesis service of Fasmac Co., Ltd. The genes *bphA1*, *bphA2*, *bphA3*, and *bphA4* encode the biphenyl dioxygenase large and small subunits, ferredoxin and ferredoxin reductase, respectively. These two fragments were cloned into the vector pUC57 (Thermo Fisher Scientific Inc., Waltham, MA, USA), respectively, and were further amplified by PCR with each plasmid as a template using PrimeSTAR HS DNA polymerase (TaKaRa Bio Inc., Shiga, Japan) and cloned into corresponding restriction sites of pET-15b (Novagen, Merck KGaA, Darmstadt, Germany) to generate plasmids pET-15b-bphA1A2(LB400) and pET-15b-bphA3A4(LB400), respectively. The primers used were as follows (underlining shows the restriction site given after the sequence): for the 2,120-bp fragment containing *bphA1A2* amplification, BphA1(GE)-pmF(Xba) (5′-ATGCATTCTAGATATTTTTTCCGCCCTGCCAAG-3′; XbaI) and BphA2(GE)-CR(Nco) (5′-ATGCATCCATGGCGTGCTGGGCTAGAAGAACAT-3′; NcoI), and for the 1,600-bp fragment containing *bphA3A4*, BphA3(GE)-NF(Nco) (5′-ATGCATCCATGGCCCAGGCGATTTAACCCTTTTA-3′; NcoI) and BphA4(GE)-CR(Nde) (5′-ATGCATCATATGCGCATCAATTCGGTTTGGC-3′; NdeI). Finally, the 1.6-kb NcoI-NdeI fragment from pET-15b-bphA3A4(LB400) was inserted into the corresponding site of pET-15b-bphA1A2(LB400) to obtain the plasmid pEA1A2A3A4(LB400), in which *bphA1A2A3A4* was under the control of the T7 promoter (Fig. 3A). The 3.7-kb XbaI-NdeI region in the resulting plasmid was sequenced, and it was confirmed that there were no unexpected mutations.

### Preparation of the E. coli cells expressing recombinant biphenyl dioxygenase from *B. xenovorans* LB400.

E. coli BL21(DE3) (Novagen) was transformed with pEA1A2A3A4(LB400) and used to express the recombinant enzyme. The E. coli cells harboring pEA1A2A3A4(LB400) were grown in 2× YT medium (1.6% Bacto tryptone, 1.0% Bacto yeast extract, 0.5% NaCl) containing ampicillin (100 µg/ml) at 30°C with shaking. When the OD_660_ of the culture reached 3.0, isopropyl-β-d-thiogalactopyranoside (IPTG) was added at a final concentration of 0.2 mM, and then culture was continued for 90 min to induce the recombinant proteins. The cells were harvested by centrifugation at 6,000 × *g* for 10 min at 15°C, washed twice with buffer A, resuspended in the same buffer, and used immediately for PCB-degrading reactions.

### PCB-degrading reaction with the composed microbial catalyst in a developed bioreactor with microbubble generation.

A 2-L bioreactor was developed for scale-up of the PCB-degrading studies. The microbubble generation procedure introduces 1 L of buffer A into a pressurized microbubble generation tank and subsequently introduces oxygen gas to apply a pressure of 0.5 MPa. There is an open/close valve in the middle of the piping connecting the tank and the vessel. Since the oxygen is dissolved in the buffer, cavitation occurs while passing through the water when the valve is opened. The oxygen is subsequently turned into hyperoxygen gas dissoluble microbubbles.

The cells of *C. testosteroni* YU14-111, grown in W-medium with biphenyl and stored at −80°C, were washed twice, resuspended in buffer A, and added into the reaction vessel through the sample injection valve system. The recombinant E. coli cells expressing 3,4-dioxygenase, which were prepared freshly as described above, were also added to the vessel through the same valve system. The cells of the E. coli and YU14-111 had an OD_660_ of 16 and 4, respectively. Then, KC-300 and Triton X-100 were injected into the vessel using the sample injection valve system at final concentrations of 40 mg/L and 0.005% (wt/vol), respectively. The reaction was carried out at 30°C with 600 rpm of the mix-rotation. At 5 min, 1 h, 3 h, 6 h, and 24 h after the injection of KC-300, about 5 mL of the reaction mixture was collected, and the remaining PCBs were analyzed as described above. The data were collected from the three repeated experiments with the bioreactor.
